# Dopaminergic Dysregulation in Migraine: From Hypothalamic A11 Dysfunction to a Systemic Biobehavioral Phenotype

**DOI:** 10.3390/ijms27167262

**Published:** 2026-08-14

**Authors:** Jakub Przegrałek, Filip Targosiński, Karol Marschollek, Izabela Łaczmańska, Lanfranco Pellesi, Sławomir Budrewicz, Marta Nowakowska-Kotas, Marta Waliszewska-Prosół

**Affiliations:** 1 Department of Neurology, Wroclaw Medical University, 50-556 Wroclaw, Poland; przegralekj@gmail.com (J.P.); filip.targosinski@umw.edu.pl (F.T.); karol.marschollek@gmail.com (K.M.); slawomir.budrewicz@umw.edu.pl (S.B.); marta.nowakowska-kotas@umw.edu.pl (M.N.-K.); 2 Department of Clinical and Experimental Pathology, Wroclaw Medical University, 50-556 Wroclaw, Poland; izabela.laczmanska@umw.edu.pl; 3 Clinical Pharmacology, Pharmacy and Environmental Medicine, Department of Public Health, University of Southern Denmark, 5230 Odense, Denmark; lanfranco.pellesi@gmail.com

**Keywords:** migraine, dopamine, A11 nucleus, hypothalamus, trigeminovascular system, neuroimaging, medication-overuse headache, reward system

## Abstract

Beyond the well-established roles of serotonin and calcitonin gene-related peptide (CGRP), the contribution of dopamine (DA) to migraine pathophysiology remains a subject of intense investigation. DA acts as a neuromodulator, functioning both as a homeostatic pain filter and a driver of premonitory and autonomic symptoms. This review provides a comprehensive synthesis of DA’s involvement in migraine, proposing an integrated model that spans from molecular signaling to a systemic phenotype. A comprehensive search of the PubMed database (1976–2026) was performed. The analysis evaluates data across receptor signaling, hypothalamic connectivity (specifically the A11 nucleus), genetic susceptibility markers and advanced neuroimaging studies. Current evidence identifies dopamine as a gatekeeper of the trigeminovascular threshold, with the hypothalamic A11 nucleus serving as a modulatory hub. Within this circuit, DA exerts concentration-dependent effects: high-affinity D2 receptor activation promotes antinociception, whereas low-affinity D1-like receptors facilitate nociceptive signaling. Clinically, this manifests as a “dopaminergic phenotype” characterized by yawning, nausea, and reward-system dysfunction. This phenotype extends beyond migraine, showing links to comorbidities such as restless legs syndrome (RLS) and attention-deficit/hyperactivity disorder (ADHD). DA’s role in migraine is state-dependent and interconnected with CGRP and serotonergic pathways. Recognizing the dopaminergic phenotype is essential for capturing the clinical heterogeneity of the disorder and may pave the way for personalized, mechanism-based therapeutic strategies.

## 1. Introduction

Dopamine (DA) is a neurotransmitter essential to both the peripheral and central nervous systems (CNS) [1,2]. Within the CNS, dopaminergic signaling orchestrates motivation, cognitive function, motor control, mood, and reward-seeking behavior [2,3]. Furthermore, it modulates autonomic reflexes, including emesis and yawning [4,5]. Dysregulation of this system is a hallmark of Parkinson’s disease, attention-deficit/hyperactivity disorder (ADHD), schizophrenia, and affective disorders [1,2,3]. Notably, conditions with dopaminergic involvement, such as restless legs syndrome and depression, exhibit high comorbidity with migraine [6,7].

Migraine remains one of the most disabling neurological disorders [8,9], ranking third globally in terms of disability-adjusted life years (DALYs) [9]. Affecting approximately 1.16 billion people, it imposes a staggering burden on global healthcare and economies [8,9,10,11]. Clinically, migraine attacks are characterized by debilitating symptoms, including nausea, vomiting, photophobia, and sensory hypersensitivity (phonophobia, osmophobia, or cutaneous allodynia) [11,12,13,14,15]. As the second most common primary headache after tension-type headache (TTH) [16], migraine is characterized by a 3- to 4-fold higher prevalence in females [17,18,19].

According to the International Classification of Headache Disorders, 3rd edition (ICHD-3), migraine is classified into migraine with aura (MwA) and migraine without aura (MwoA) [17]. MwA is distinguished by transient neurological symptoms (aura) affecting visual, sensory, speech, or motor functions [17,20]. Depending on frequency, migraine is further categorized into episodic (EM) and chronic (CM), the latter defined by 15 or more headache days per month [17,21].

A migraine attack typically progresses through four phases: (1) the prodrome (premonitory phase), (2) the aura, (3) the headache phase, and (4) the postdrome [22,23,24]. The prodrome begins hours or days before the pain and includes symptoms such as food cravings, polyuria, mood changes, yawning, and nausea [23,23,25]. The subsequent headache is unilateral, pulsating, and aggravated by physical activity [17,21]. Finally, the postdrome often involves fatigue and impaired concentration [26,27]. Notably, non-headache symptoms frequently overlap throughout the ictal period, suggesting a common underlying mechanism [14,28,29,30,31,32,33].

While the exact pathophysiology of migraine remains partially elusive, historical focus has centered on serotonergic pathways and calcitonin gene-related peptide (CGRP) [13,14,21]. As early as the 1970s, Sicuteri proposed that dopamine plays a pivotal role, particularly through receptor hypersensitivity [34]. This hypothesis has been reinforced by the clinical efficacy of dopamine antagonists in acute migraine treatment [35]. Contemporary findings regarding the hypothalamic A11 nucleus, which modulate pain sensitization via descending dopaminergic pathways, strengthen the DA’s importance in migraine [36,37,38]. Despite accumulating evidence, a framework capturing all facets of dopaminergic involvement in migraine is still lacking.

The aim of this narrative review is to comprehensively examine the role of the dopaminergic system in migraine. This article analyzes the mechanisms of DA in migraine pathophysiology, evaluates dopamine-mediated clinical symptoms, and discusses potential implications for future therapeutic strategies.

## 2. Methods

A comprehensive literature search was conducted via the PubMed database to investigate the relationship between the dopaminergic system and migraine. The search utilized the following Boolean string: *(migraine OR “migraine patients” OR “migraineurs” OR “chronic migraine” OR “episodic migraine” OR “migraine population”) AND (dopamine OR “dopaminergic system” OR “dopamine receptors” OR “dopamine agonists” OR “dopamine signaling” OR “dopamine dysfunction” OR “dopamine antagonists”)*. The search covered publications from 1976 to 15 April 2026, yielding 561 records. After identifying and removing two duplicate entries, 559 records remained for screening.

A secondary, targeted search was performed to identify dopamine neuroimaging studies using the phrase: *(migraine OR “migraine patients” OR “migraineurs” OR “chronic migraine” OR “episodic migraine” OR “migraine population”) AND (dopamine OR “dopaminergic system” OR “dopamine receptors” OR “dopamine agonists” OR “dopamine antagonists”) AND (PET OR SPECT OR neuroimaging OR imaging)*. This yielded 53 additional records for screening.

Both original research articles and reviews were included in the analysis based on their clinical and pathophysiological relevance. Exclusion criteria comprised conference abstracts, editorials, letters, comments, and non-English language publications. Following a title and abstract screening, approximately 200 articles were deemed relevant. Furthermore, foundational studies and pertinent sources identified through manual searching of reference lists (snowballing) were included to ensure a comprehensive synthesis of the literature.

## 3. Genetic Insights into the Dopaminergic System and Migraine

The advent of Next-Generation Sequencing (NGS), coupled with advanced computational models, has reshaped our understanding of the genetic architecture of migraine. Early candidate-gene studies focused on single polymorphisms within the dopaminergic system, including *DRD2*, *DRD4*, *DAT1 (SLC6A3)*, *DBH*, *COMT*, and *MAOA* [39,40]. However, these isolated approaches are now considered insufficient in the era of large-scale genomic analyses. Current evidence indicates that migraine is a complex polygenic disorder characterized by intricate interactions between neuronal, vascular and neurotransmitter systems [41,42,43,44]. Despite technical advancements, the precise signaling networks and genetic mechanisms underlying susceptibility remain elusive.

Recent genome-wide association studies (GWAS) and meta-analyses, involving hundreds of thousands of participants, have identified over 120 migraine-associated loci [42,43]. Most of these loci do not directly map to classical dopaminergic genes, but evidence suggests that altered dopaminergic neurotransmission contributes to pathophysiology by modulating trigeminovascular signaling and pain-processing pathways [45]. Furthermore, monogenic migraine syndromes, such as Familial Hemiplegic Migraine (FHM), demonstrate that pathogenic variants in *CACNA1A*, *ATP1A2*, and *SCN1A* disrupt ion homeostasis and synaptic transmission. These disruptions may impair dopaminergic signaling, acting as modifiers of migraine susceptibility and treatment responsiveness [44,46]. Large-scale multi-omics analyses further suggest that migraine susceptibility is driven by interconnected molecular pathways. Disturbances in dopamine-mediated signaling may further contribute to disease chronification, central sensitization, and the heterogeneity in therapeutic response [47,48].

### Epigenetic Mechanism Involved in Migraine Pathophysiology

Epigenetic regulation through microRNAs may contribute to migraine biology and treatment effects [49,50]. MicroRNAs regulate gene expression post-transcriptionally and have been proposed as biomarkers, although evidence does not support diagnostic, prognostic, or predictive use [49]. In pediatric migraine without aura, salivary miR-34a-5p and miR-375 levels were higher in untreated patients than controls, whereas serum and salivary levels were lower during NSAID and magnesium treatment [50]. Of dopaminergic relevance, miR-34a-5p has been reported in dopaminergic circuits, while in silico analyses suggested that both microRNAs target pathways involved in pain transmission and soluble-factor regulation [50]. However, this pilot study cannot establish dopamine-specific mechanisms in migraine [50]. In women receiving erenumab, miR-143-3p decreased with greater treatment response in episodic migraine, while elevated miR-34a-5p and miR-382-5p in chronic migraine declined after treatment [51]. In medication-overuse headache, preliminary evidence indicates low-grade inflammation, differential DNA methylation in immune- and pain-related genes, and altered regulatory T-cell and microglial signaling [52]. However, this evidence does not establish direct microRNA and dopamine-specific involvement [52]. Thus, microRNAs offer a plausible link to dopaminergic migraine mechanisms, but due to the sparse evidence, this relationship remains uncertain and requires future extensive research.

## 4. Neurochemical and Receptor-Level Mechanisms of Dopaminergic Involvement in Migraine

In 1977, Sicuteri proposed that the dopaminergic system is a critical factor in migraine etiology, second only to serotonergic dysfunction. This seminal hypothesis initiated a trajectory of research focused on DA’s precise role. Over subsequent decades, several mechanisms have been explored, including DA receptor hypersensitivity [53,54], altered catecholamine metabolism [55], and the involvement of the hypothalamic A11 nucleus in modulating migraine symptoms [56]. Methodological advancements have since enabled a more granular assessment of the dopaminergic system, providing robust evidence of its significance. This section evaluates these hypotheses in light of contemporary clinical and experimental findings.

### 4.1. Hypothesis 1: Dopaminergic Hypersensitivity in Migraine

The leading hypothesis suggests that migraineurs exhibit receptor hypersensitivity to DA [53,57,58]. This concept arises from two observations: the high prevalence of migraine symptoms mediated by dopaminergic pathways (e.g., yawning, nausea) [5,59,60,61] and pharmacological evidence showing that DA agonists can induce premonitory-like symptoms in patients [53]. Indirect support is provided by the clinical efficacy of DA antagonists in managing acute attacks [62].

Studies from the 1990s and early 2000s reported heightened sensitivity to dopaminergic agents, manifested by exaggerated prolactin responses [63], induced yawning [64,65] and various non-headache symptoms [64,66]. Piccini et al. also demonstrated that apomorphine administration induced significant cerebral artery dilation in individuals with MwoA compared to healthy controls and TTH patients [67]. These early studies often utilized unblinded designs and small cohorts.

#### 4.1.1. The “Fluctuation Model” and Receptor Regulation

The mechanisms driving this putative hypersensitivity remain partially elucidated. Barbanti et al. hypothesized that chronic DA depletion triggers the upregulation of pre- and postsynaptic D1-like and D2-like receptors [68]. In this model, a surge in DA levels prior to the headache phase activates these upregulated receptors, precipitating prodromal symptoms. During the postdrome, DA levels decline but continue to stimulate sensitized receptors, contributing to lingering symptoms [59,59,68].

Supporting this “fluctuation” model, Castillo et al. found significantly elevated cerebrospinal fluid levels of 3,4-dihydroxyphenylacetic acid (DOPAC), the primary DA metabolite, following migraine episodes, suggesting a period of high dopaminergic activity [69]. Functional MRI (fMRI) data also indicate increased activation of the substantia nigra during attacks [70]. Regarding receptor density, increased density of D3, D4, and D5 receptors has been identified on lymphocyte membranes in migraineurs, potentially reflecting systemic upregulation [71,72]. Some studies noted increased affinity, rather than density, of D2 receptors in peripheral platelets [73], while others observed reduced interictal visual attention in children, suggesting that low dopamine levels between attacks may impair cognitive performance [74].

Elevated ictal DA may also result from impaired conversion of DA to norepinephrine (NE) by dopamine beta-hydroxylase (DBH). Some studies found lower interictal DBH activity in MwA and MwoA patients [75]. An initial surge in the substrate (DA) may temporarily overstimulate the enzyme. Although various *DBH* gene polymorphisms have been linked to migraine [76,77,78,79,80,81,82], conflicting results persist [39,83]. Interestingly, the DBH 19-bp insertion/deletion polymorphism may specifically predispose patients to medication overuse in chronic migraine [84].

#### 4.1.2. Dopamine and Cortical Spreading Depression (CSD)

A potential trigger for the ictal DA elevation involves cortical spreading depression (CSD). Chen et al. [57] proposed that interictal dopaminergic hypofunction facilitates CSD initiation, which subsequently triggers glutamatergic outputs that stimulate subcortical dopaminergic neurons. This relationship appears bidirectional: animal models suggest DA modulates CSD via D1 and D2 receptors [85,86]. Specifically, D2 antagonists decrease CSD amplitude and duration, while D2 agonists exert the opposite effect [86]. However, a recent study by Guerrero Prieto et al. showed that quinpirole (D2 and D3 receptors agonist) only temporarily increased the CSD propagation, ultimately suppressing it with exposure time [87]. These findings suggest that dopamine modulation on CSD propagation might be time- or dose-dependent.

#### 4.1.3. Molecular Imaging

A PET study by DaSilva et al. [88] found that the binding potential (BP) of [11C]raclopride (a D2/D3-selective radioligand) increased in the striatum during attacks. Since [11C]raclopride competes with endogenous dopamine, this increase suggests *decreased* striatal DA release during the ictal phase, challenging the “hyperdopaminergic” hypothesis for this specific region. Conversely, BP decreased in the insula during attacks [88].

Ultimately, many mechanisms remain elusive. Alterations in DA transmission may be secondary to disruptions in the serotonergic [58] or noradrenergic systems [89]. These networks interact extensively and their complex crosstalk remains a subject for further investigation.

### 4.2. Hypothesis 2: Hypothalamic Descending Pathways of the A11 Nucleus Are Involved in Migraine

The hypothalamic and thalamic nuclei are considered pivotal in modulating, and potentially generating, migraine [36,38,90]. This hypothalamic involvement is largely mediated by the dopaminergic projections of the A11 nucleus, which connects to several pain centers, including the thalamus, the rostral dorsal pons, and the trigeminocervical complex (TCC) in the medulla [38,91,92]. The A11 nucleus, a group of tyrosine hydroxylase (TH)-positive neurons in the posterior hypothalamus, serves as the primary source of dopamine for the spinal cord [93].

In animal models, dopamine exerted a potent inhibitory effect on TCC neurons, likely mediated via D1-like or D2-like receptors expressed within the complex [94]. Subsequent research confirmed that D2 agonists produce similar antinociceptive effects, whereas D2 antagonists counteract this inhibition, suggesting that dopamine suppresses TCC firing primarily through D2 signaling [95,95]. Charbit et al. further established the A11-TCC link in migraine. In a rat model of dural stimulation, electrostimulation of the A11 nucleus inhibited migraine-evoked potentials in the TCC, an effect reversed by D2 antagonists. D2 receptors were primarily found on cell bodies (suggesting a postsynaptic inhibitory role), while D4 receptors were located on nerve endings, likely serving presynaptic autoregulatory functions [96].

**Figure 1:**
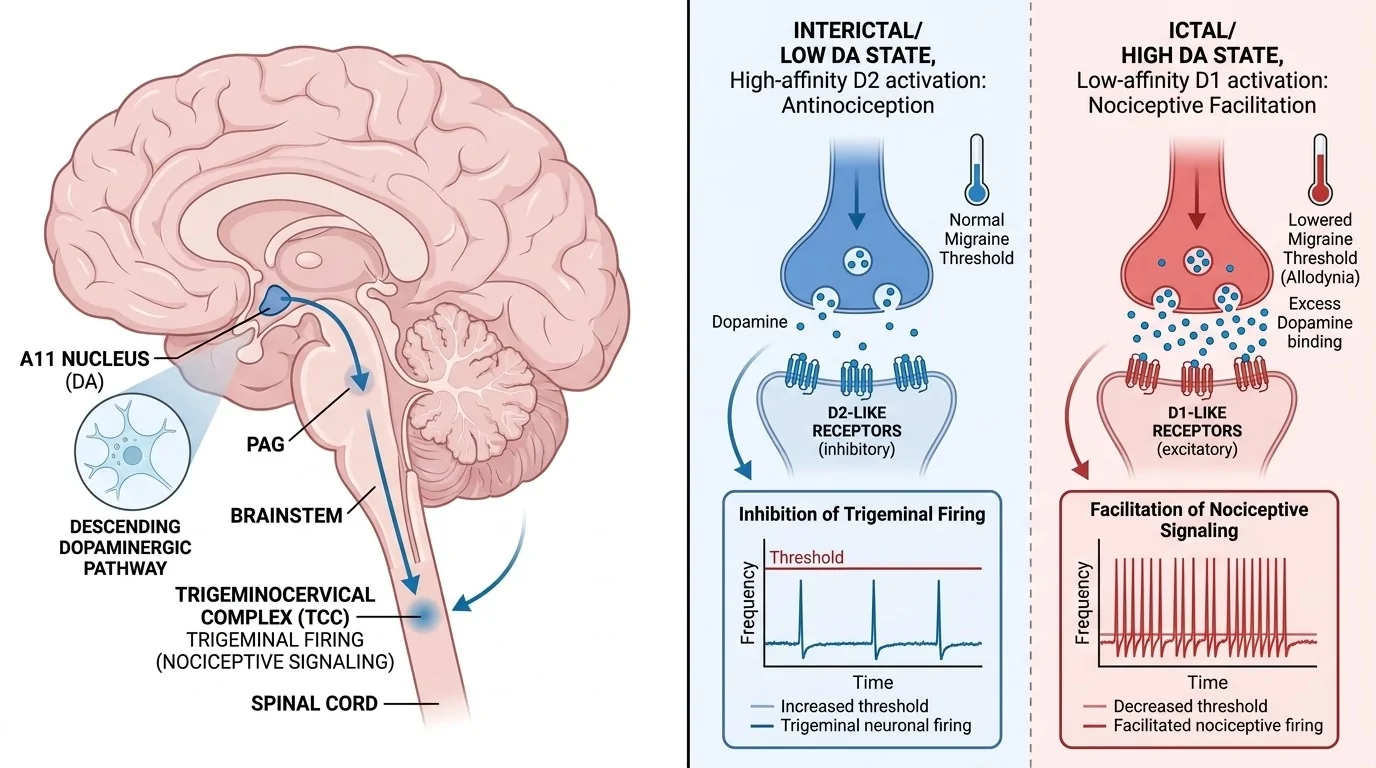
The hypothalamic A11-trigeminovascular axis and concentration-dependent dopaminergic modulation in migraine.

Newer work by Li et al. [37] established a nuanced “dose-dependent” model involving both D1 and D2 receptors. In their study, ablation of the A11 nucleus lowered pain thresholds and exacerbated photophobia in acute and chronic migraine mice. A11 fibers interacted with both D1- and D2-positive neurons. According to their model, low concentrations of dopamine preferentially bind to D2 receptors, exerting an anti-migraine effect. However, as DA levels rise, the subsequent activation of D1 receptors abolishes this inhibition, potentially promoting nociception. This dopaminergic release in the A11 is further modulated by paracrine GABAergic transmission [37,97,98] (Figure 1).

The opposing effects of D1 (pro-nociceptive) and D2 (anti-nociceptive) receptors are consistent with some literature [98,99,100], but they appear to contradict the therapeutic efficacy of central D2 antagonists. For instance, D2 antagonists alleviated nitroglycerin-induced allodynia and reduced CGRP release, while the agonist apomorphine aggravated symptoms [101]. These discrepancies may stem from the route of administration: Baranoglu Kilinc et al. utilized systemic (intraperitoneal) injections, whereas Li et al. employed direct microinjections into the trigeminal nucleus caudalis (TNC) [37,101]. Thus, dopaminergic modulation may exert divergent effects at different levels of CNS. Furthermore, differences in the chemical properties and receptor affinities of the agents used (e.g., non-selective apomorphine vs. selective agonists) complicate direct comparisons.

Despite these insights, evidence for the A11 nucleus’s role in migraine is currently exclusively preclinical. Due to the lack of clinical data in humans, the model remains largely hypothetical and may be relevant only to animals. It should also be noted that animal models of migraine are limited by a substantial translation gap, as animals cannot self-report headache, and many of the outcome measures used are merely indirect indicators of trigeminovascular system activation. Moreover, the neurochemical profile of A11 neurons remains a subject of debate, with some data suggesting that they lack the dopamine transporter (DAT) and may primarily synthesize L-DOPA rather than dopamine [102]. Koblinger et al. proposed that A11 neurons might release dopamine via non-traditional mechanisms [103], but the functional heterogeneity of these neuronal groups requires further investigation [104].

### 4.3. Hypothesis 3: Dysfunctional Tyrosine Metabolism Leads to Disruption of Elusive Amines and Dopamine

**Figure 2:**
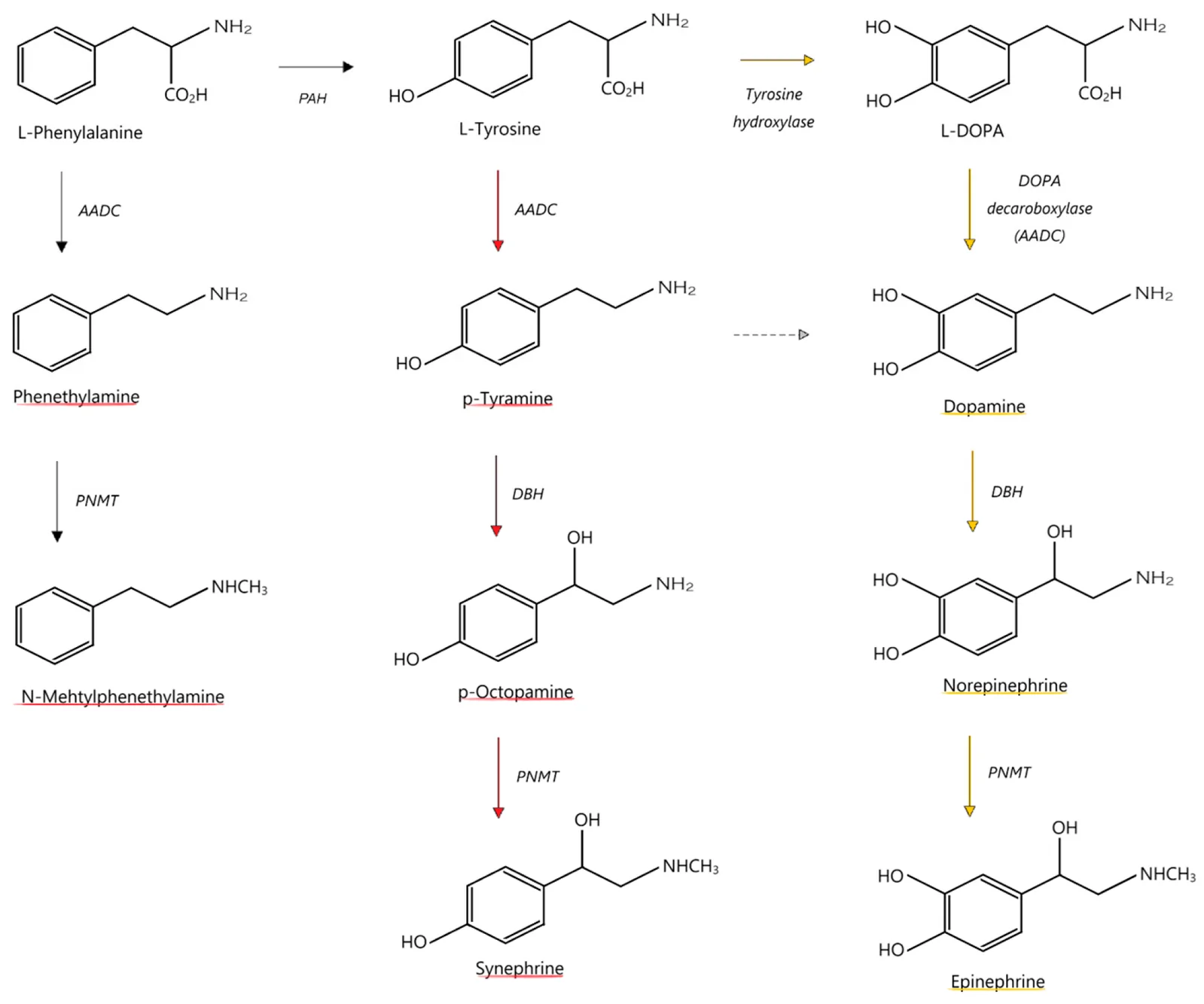
Endogenous biosynthetic pathways of catecholamines and selected trace amines derived from L-phenylalanine and L-tyrosine. Yellow arrows indicate the major catecholamine biosynthetic pathway, leading from L-tyrosine to L-DOPA, dopamine, norepinephrine, and epinephrine. Red arrows represent alternative L-tyrosine metabolic pathways leading to the formation of trace amines. The dashed arrow denotes a minor pathway contributing to dopamine biosynthesis. Trace amines are indicated by red underlining, while catecholamines are marked with yellow underlining. **Abbreviations:** AADC, aromatic L-amino acid decarboxylase; DBH, dopamine β-hydroxylase; L-DOPA, L-3,4-dihydroxyphenylalanine; PAH, phenylalanine hydroxylase; PNMT, phenylethanolamine N-methyltransferase.

D’Andrea et al. were the first to propose that tyrosine metabolism is altered in migraineurs, leading to the accumulation of trace amines (TAs) and subsequent fluctuations in dopamine levels [55,105,106]. Trace amines are a group of endogenous compounds, structurally analogous to classical monoamines, that exert neuromodulatory functions within the central nervous system (CNS) [107]. In the human brain, their physiological concentrations are significantly lower (typically in the nanomolar range) than those of primary biogenic amines like DA or serotonin [108,109,110]. Their biological actions are mediated through a dedicated class of G protein-coupled receptors known as trace amine-associated receptors (TAARs), with TAAR1 being the most prominent mediator of these interactions [107,111,112]. TAs are derived from tyrosine and phenylalanine, including tyramine (Tyr), octopamine (Oct), synephrine (Syn) and phenylethylamine (PEA), and share overlapping metabolic pathways with classical catecholamines, such as dopamine and norepinephrine [108]. The biosynthetic integration of these specific TAs is illustrated in Figure 2. TAs are increasingly recognized as “rheostats” that fine-tune dopaminergic and noradrenergic signaling [113], but the functional interplay between dopamine and the trace amine system in migraine remains multifaceted and complex.

The hypothesis concerning the influence of trace amines (TAs) on migraine pathophysiology suggests that the imbalance in TA and DA concentrations stems from a metabolic shift in tyrosine processing, potentially driven by an upregulated aromatic L-amino acid decarboxylase (AADC) activity [114]. D’Andrea et al. further suggested that this enzymatic shift may be linked to underlying mitochondrial dysfunction [105]. Notably, amine profiles appear to diverge across migraine subtypes. In an early study, patients with MwoA exhibited elevated platelet concentrations of octopamine (Oct), whereas those with MwA showed higher synephrine (Syn) levels, with no significant alterations in Oct or tyramine (Tyr) [115]. Conversely, a subsequent study identified significantly elevated plasma levels of Syn and Oct in MwoA patients, while no significant differences were found in the MwA group. Furthermore, individuals with chronic migraine (CM) displayed increased platelet Tyr levels, alongside significantly elevated thrombocyte concentrations of dopamine and norepinephrine [116]. Similar findings for norepinephrine have been reported in episodic migraine [117], but results for dopamine remain conflicting [117,118]. Moreover, the observed elevations in norepinephrine partially challenge the traditional view of interictal noradrenergic hypofunction in migraineurs [89,119,120].

Despite these findings, research on TAs in migraine remains sparse. Most available evidence relies on platelets as a peripheral model for assessing TA and monoamine levels. Platelets are frequently utilized as integrative markers because they do not synthesize DA, norepinephrine or TAs de novo; instead, they sequester these amines from the systemic circulation for storage [115,118,121]. However, this model is inherently susceptible to confounding variables, such as physical activity, acute stress, platelet turnover, and activation status [121,122]. Consequently, the evidence for altered TA signaling in migraineurs remains inconclusive. There is a clear need for large-scale, replicative studies to definitively confirm or refute the role of the TAs in migraine pathogenesis.

### 4.4. Dopaminergic System Involvement in Chronic Migraine

Recent data from animal models for chronic migraine (CM) offer further insights into the role of dopaminergic dysfunction in disease progression. Zhang et al. demonstrated that dopamine levels in the prefrontal cortex were significantly lower in CM rats compared to sham controls [123]. The transition from episodic to chronic migraine may involve a fundamental biochemical shift, wherein the ictal release of dopamine, characteristic of episodic cases, eventually fails due to the depletion of intracellular stores. Moreover, a study by Yang et al. found that in CM model rats the expression of D2 receptors in the hippocampus was lower, which resulted in the activation of microglia, enhancement of neuroinflammatory pathways and, consequently, deficits in spatial and recognition memory [124].

Furthermore, preclinical studies investigating dopaminergic signaling within the trigeminal nucleus caudalis (TNC) have highlighted the role of the D2 receptor in modulating central sensitization [125,126]. Specifically, D2 receptor activation appears to influence the cellular expression and trafficking of AMPA receptor subunits, namely GluA1 and GluA2. In these models, D2 agonists alleviated allodynia by inhibiting GluA1 membrane trafficking and preventing GluA2 endocytosis. At the cellular level, this mechanism resulted in diminished intracellular calcium influx and reduced production of reactive oxygen species (ROS) [125,126]. These findings provide a rationale for the potential therapeutic application of D2 agonists in the management of chronic migraine.

### 4.5. Role of Dopaminergic Mechanisms in Vestibular Migraine

Dopaminergic mechanisms may also contribute to vestibular migraine, although the evidence remains indirect. Vestibular migraine is characterized by the presence of vestibular symptoms, such as nausea, vomiting, dizziness, or autonomic symptoms [17,127] which partly overlap with the proposed dopaminergic symptoms observed in migraine [59,68]. The experimental data indicate that dopamine can modulate medial vestibular nucleus neurons through D2-dependent presynaptic effects on GABAergic transmission and direct postsynaptic actions [121]. Moreover, some dopaminergic agents have been used to treat vestibular migraine but literature studies directly assessing their efficiency in this type of migraine are lacking [128]. Furthermore, the status of vestibular migraine as a distinct type of migraine is not clear, and the validity of this diagnosis remains a matter of debate, despite its official inclusion in the ICHD-3 classification [127,129]. It is often suggested that because of a lack of clear pathognomonic markers, vestibular symptoms may reflect migraine aura or another vestibular disorder rather than a distinct syndrome [129,130]. Therefore, although dopaminergic mechanisms may be involved in the pathophysiology of vestibular migraine, their role remains unconfirmed.

### 4.6. Summary and Synthesis of Neurochemical and Receptor-Level Hypotheses

The intersection of current theories lies at the junction of receptor hypersensitivity and altered tyrosine metabolism. The preferential synthesis of TAs over classical catecholamines may result in chronically low interictal DA levels, subsequently triggering a compensatory upregulation of dopamine receptors. This hypersensitivity likely extends to the A11-mediated modulation of the trigeminocervical complex (TCC). As proposed by Li et al., dopaminergic modulation within the TCC is concentration-dependent: during the ictal phase, the release of dopamine activates these upregulated (and potentially low-affinity) D1 receptors, thereby facilitating sensitization to nociceptive stimuli [37].

Clinical investigations of dopamine levels in migraineurs have yielded conflicting results [88,117,118,131]. Nagel-Leiby et al. identified significantly higher plasma DA levels in women with MwoA compared to healthy controls, though this was observed exclusively during menstruation. Similarly, platelet-based studies produced divergent outcomes: one study showed no difference in thrombocyte DA concentrations between groups [117], while another reported significantly elevated levels in MwoA subjects [118]. These discrepancies likely stem from methodological heterogeneity and the inherent limitations of the platelet model as a proxy for central neurotransmission.

The primary limitation of the hypersensitivity hypothesis is its “unitary” framework. Given the complexity of the dopaminergic system, characterized by multifaceted regulation and high regional specificity [2,132,133,134], it is improbable that global fluctuations in CNS dopamine would result in such a selective clinical phenotype. It is more plausible that dopaminergic alterations are region-specific, with the hypothalamus and TCC serving as the most likely sites of dysfunction. The hypothalamus has been frequently proposed as a central hub in migraine pathogenesis [57,132,135], but direct evidence remains sparse. Given the hypothesized role of the A11-TCC axis in pain [37,56,94,95,96], well-designed human neuroimaging studies are essential to validate these preclinical models.

In conclusion, dopamine is integral to both the cephalic and systemic manifestations of migraine, but current frameworks do not fully capture the disorder’s complexity. Furthermore, the crosstalk between DA and other key mediators, such as CGRP and serotonin, remains largely unmapped. Since much of the foundational research dates to the late 20th and early 21st centuries, there is an urgent need for contemporary studies utilizing molecular and imaging techniques to resolve these enduring questions.

## 5. Dopaminergic Crosstalk in Migraine Pathophysiology

### 5.1. Dopamine–Serotonin–CGRP Interactions

CGRP signaling within the trigeminovascular system is a cornerstone of migraine pathophysiology. Released from peripheral and central trigeminal afferents, CGRP promotes meningeal vasodilation, neurogenic inflammation, and the facilitation of nociceptive transmission within the trigeminocervical complex (TCC) [45,136,137]. Serotonergic mechanisms are well-established in this context and dopaminergic signaling is recognized as a significant, albeit less defined, modulator of trigeminal activity and CGRP release [137,138].

Experimental data suggest that dopamine influences trigeminovascular processing at multiple levels, including the trigeminal ganglion and second-order neurons in the TCC. Dopamine receptors, categorized into the D1-like and D2-like families, exert opposing effects on intracellular cyclic AMP (cAMP) [96,139]. Since CGRP secretion is dependent on calcium influx and downstream second-messenger pathways, dopaminergic input may indirectly shape neuronal excitability. Specifically, activation of D2-like receptors is associated with reduced neuronal firing and decreased neurotransmitter release; conversely, a reduction in dopaminergic inhibitory tone may facilitate trigeminal nociceptive signaling [139,140].

More speculative is the potential interaction between dopamine and other sensory modulators, such as transient receptor potential vanilloid 1 (TRPV1) channels and endocannabinoid pathways. Although supported by preclinical models, the clinical relevance of these interactions to CGRP in humans remains uncertain [137,139,141].

In contrast, serotonergic modulation of CGRP is supported by robust clinical evidence. Activation of 5-HT1B/1D receptors inhibits CGRP release from trigeminal terminals, representing the mechanism of triptans [45,142]. Beyond these, 5-HT3 receptors may influence the monoaminergic balance, including dopaminergic signaling. However, their direct contribution to CGRP regulation is less established [137,143].

The convergence of serotonergic and dopaminergic systems within migraine-relevant circuits suggests a functional interplay between the TCC and its hypothalamic connections. Both D2-like and 5-HT1 receptors signal primarily through Gi-coupled pathways, leading to convergent effects on cAMP levels and neuronal excitability [140].

These pathways may explain the significant heterogeneity of migraine phenotypes. Dopaminergic dysfunction is associated with premonitory and autonomic symptoms such as yawning, nausea, somnolence, and hypotension, whereas CGRP-driven activation is more linked to the headache phase and sensory hypersensitivity. Serotonergic pathways may function as an intermediate regulatory layer, modulating both trigeminal excitability and broader brainstem dynamics [45,68].

**Figure 3:**
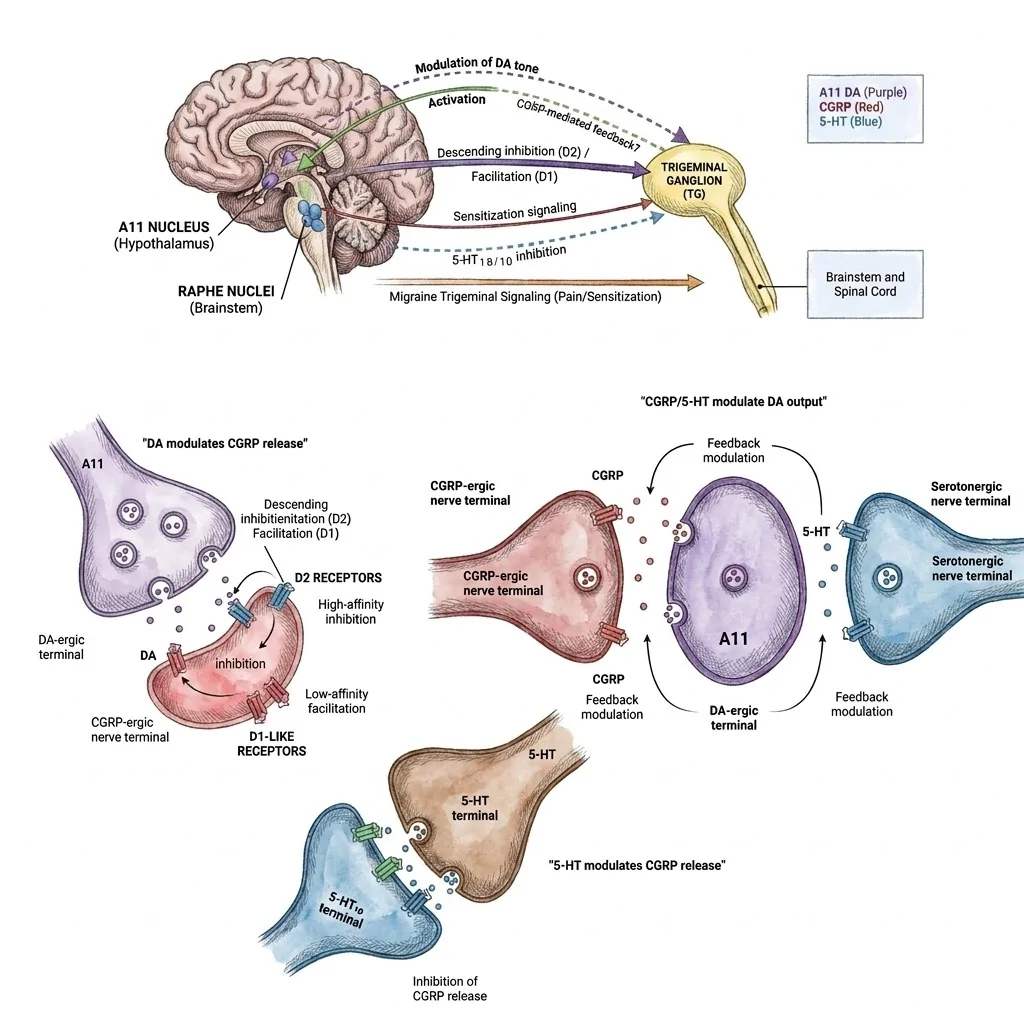
The trilateral neuroendocrine circuit in migraine.

Current evidence supports the existence of an interconnected network involving dopaminergic, serotonergic, and CGRP systems [144] (Figure 3). Serotonin exerts a well-defined inhibitory influence over trigeminovascular CGRP signaling and dopaminergic effects appear more modulatory and state-dependent. The precise characterization of receptor-specific interactions within these pathways may further elucidate migraine heterogeneity and guide the development of targeted, personalized therapeutic strategies [45,139].

### 5.2. Influence of Sex Hormones

Migraine in women is characterized by a higher prevalence and distinct clinical phenotypes across the lifespan. The threefold predominance of the disorder in females after puberty suggests a pivotal role for sex hormones, particularly estrogens, in disease expression and susceptibility [145]. Estrogens are considered a main neuromodulatory factor influencing migraine susceptibility through changes in neuronal excitability and sensory processing within trigeminovascular and brainstem networks [137,145]. Estrogen interacts with multiple neurotransmitter systems, including serotonergic, dopaminergic and glutamatergic pathways, thereby modulating pain processing and autonomic responses [145,146]. Its receptors are expressed in migraine-relevant components of the trigeminal system and central nervous system, providing a molecular basis for hormone-dependent modulation of nociceptive signaling [147]. Estrogen may also act as a key regulator of 5-HT signaling and cortical excitability, shifting the migraine threshold across different hormonal states [142,145,148]. The role of progesterone remains less clearly defined. It is suggested that progesterone may exert modulatory effects by attenuating neurogenic edema and mast-cell histamine release, reducing trigeminal nociceptive activity, and limiting prostaglandin synthesis [147,149].

Current evidence indicates that fluctuations in ovarian hormone levels, especially estrogens, may be more relevant to migraine susceptibility than their absolute concentrations, providing a framework for understanding the marked variation in migraine across the menstrual cycle and reproductive life [147,150]. The estrogen withdrawal hypothesis proposed by Somervilles linked the rapid decline in estradiol during the late luteal phase with the increased occurrence of menstrual migraine [151,152,153]. Estrogen withdrawal may increase trigeminovascular susceptibility and influence neurotransmitter and neuropeptide signaling, thereby facilitating attack initiation [147]. For these reasons perimenstrual migraine attacks are associated with greater disability and may be less responsive to acute treatment than attacks occurring outside the perimenstrual period [154]. On the other hand, relatively stable hormonal conditions are frequently associated with improvement in migraine. This is illustrated by the milder migraine course experienced by many women, particularly those with migraine without aura, during the pregnancy [141,143,144]. Migraine also improves in many women after natural menopause, when hormone concentrations become chronically low and relatively stable, whereas perimenopause represents another period of increased migraine burden because of marked fluctuations in ovarian function and estrogen levels [147,149,155]. However, these effects are not uniform between individuals and may be influenced by age, migraine subtype, and other biological characteristics [149,155].

Dopaminergic sensitivity also appears to be under hormonal control, affecting the expression of the migraine phenotype. Experimental studies indicate that estrogens alter D2 receptor binding and dopamine release within striatal and hypothalamic regions [156]. This modulation likely underlies sex-related differences in the prevalence and intensity of nausea, yawning and other autonomic features reported more frequently by women. Nausea, one of the most disabling non-pain symptoms, may reflect sex-dependent sensitization of brainstem emetic circuits, such as the area postrema, via its dopaminergic inputs [156]. Similarly, yawning is a premonitory marker mediated by D2/D3 signaling in the hypothalamus that supports the involvement of dopaminergic activation in the early phases of an attack [68,139].

## 6. Dopaminergic “Phenotype” of Migraine: Clinical Aspects

There is an ongoing debate regarding whether dopaminergic involvement in migraine is reflected by a specific clinical presentation. In the existing literature, symptoms that are frequently associated with the dopaminergic component include yawning, somnolence, nausea, vomiting, fatigue, mood fluctuations, and polyuria [68].

These symptoms can manifest across all phases of a migraine attack, including the prodromal, ictal and postdromal periods, with significant interindividual variability [14]. In some patients, these features are discrete, irregular, and difficult to characterize; in others, they form a consistent and distinct pattern. This observed heterogeneity supports the concept of a “dopaminergic phenotype” (or endophenotype) of migraine. This subtype is potentially driven by dopaminergic dysregulation and is characterized by prominent, reproducible non-pain symptoms that are temporally linked to the attack (see Table 1).

### 6.1. Prodromal Symptoms

The prodromal phase encompasses symptoms that precede the headache and signal the onset of an attack. These symptoms can emerge between a few hours and up to 48 h before the headache phase, presenting significant heterogeneity [68].

In a large cross-sectional study of 1148 patients, at least one dopaminergic symptom was observed in 32.6% of the population [59]. The most prevalent symptoms were yawning (63.6%), somnolence (57.5%), nausea (51.3%), vomiting (23.0%), fatigue (23.0%), mood changes (7.0%), and diuresis (3.5%). These symptoms occur throughout all phases, but 33.7% of patients reported them specifically during the prodrome. Furthermore, the presence of dopaminergic features was independently associated with longer attack duration, osmophobia, allodynia, and unilateral cranial autonomic symptoms.

Consistent with these findings, Laurell et al. identified yawning as the most frequent premonitory symptom, reported by 34% of 2714 participants [158]. Women experienced these symptoms more frequently than men and a higher migraine burden correlated with a greater number of prodromal features. Electronic diary studies have provided further insights; for instance, Giffin et al. found that patients most reported feeling tired and weary (72%), difficulty concentrating (51%) and neck stiffness (50%), with patients correctly predicting an attack in 72% of cases where prodromal symptoms were present [159]. Similarly, Schoonman et al. (2006) reported at least one premonitory symptom in 86.9% of respondents, with fatigue (46.5%), phonophobia (36.4%) and yawning (35.8%) being the most prevalent [160].

Recent data from the PRODROME trial [161], based on 4802 electronic diary entries from 920 participants, highlighted photophobia (57.2%), fatigue (50.1%), neck pain (41.9%), phonophobia (33.9%), and cognitive difficulties (30.0%) as leading symptoms. A meta-analysis confirmed this prevalence, estimating that at least one prodromal symptom occurs in 29% of population-based samples and 66% of clinical-based cohorts [25].

However, the reported frequency of these symptoms may be subject to methodological bias. The REFORM study [33] demonstrated that the prevalence of premonitory symptoms increased from 43.0% when using open-ended questions to 69.9% when patients were presented with a predefined list. Structured questionnaires may be necessary to capture the full spectrum of the dopaminergic phenotype, as patients may not spontaneously associate subtle symptoms like fatigue or concentration difficulties with their migraine attacks.

### 6.2. Headache Phase

In the headache phase, the clinical expression of the dopaminergic phenotype is characterized by gastrointestinal and autonomic symptoms, most notably nausea and vomiting. These manifestations are central to the migraine profile, as they are included in the ICHD-3 diagnostic criteria and rank among the most frequent symptoms accompanying the pain phase [17].

Within the dopaminergic spectrum, nausea and vomiting are strongly associated with the ictal period, whereas yawning and somnolence predominate during the prodrome, and polyuria or mood shifts often characterize the postdrome. However, these phases are not mutually exclusive; symptoms frequently overlap and may persist throughout the attack, especially when they form a consistent, individualized clinical pattern [68].

Data from the American Migraine Prevalence and Prevention (AMPP) Study, involving 6488 individuals with episodic migraine, revealed that 49.5% of respondents experienced headache-related nausea in at least half of their attacks, while only 6.9% never experienced it [162,163]. This high prevalence is corroborated by other research: Gajria et al. reported nausea in approximately 73% and vomiting in 29% of patients [157]. Furthermore, a population-based study [164] found that 61.8% of participants identified nausea as their “most bothersome symptom”, ranking it significantly higher than phonophobia (25.3%), vomiting (10.0%) and photophobia (2.9%).

The clinical impact of these symptoms is profound. Togha et al. observed that the presence of visceral-autonomic symptoms during an attack correlates with increased headache duration and severity [165]. Similarly, the AMPP analysis demonstrated that frequent nausea is independently associated with a higher disease burden, greater disability and increased healthcare resource utilization [163].

### 6.3. Postdromal Symptoms

The postdrome is the final stage of a migraine attack, encompassing symptoms that emerge or persist after the headache has subsided. According to the ICHD-3, postdromal symptoms can last for up to 48 h [17]. Fatigue and cognitive difficulties are the most frequently reported features, while euphoria and polyuria are other symptoms potentially linked to the dopaminergic component [68].

Data regarding the postdromal phase remains relatively sparse compared to other phases. In a prospective electronic diary study, 81% of patients reported at least one non-headache symptom during the postdrome. Fatigue or weariness was the most prevalent, occurring in 88% of episodes, followed by difficulty concentrating (56%) and neck stiffness (42%). Interestingly, nausea persisted in the postdrome for many patients. Most individuals returned to normal functioning within 24 h after the resolution of the headache [27]. Similarly, Karsan et al. demonstrated that postdromal symptoms occurred in nearly all participants during both nitroglycerin-induced and spontaneous attacks observed in a laboratory setting, with fatigue, hunger, and mood changes being the most prominent features [30].

As with the prodromal phase, objectifying postdromal symptoms remains a significant challenge. In the REFORM study of 631 migraine patients, postdromal symptoms were reported by 66.7% of respondents through open-ended questioning, but this proportion increased to 80.7% when a prompted symptom list was utilized [32]. This discrepancy highlights the substantial influence of data collection methods on reported frequencies. A systematic review and meta-analysis confirmed that postdromal symptoms are common, with fatigue (55%), concentration difficulties (35%) and mood changes (29%) being the most frequent [26]. The authors noted that the quality of evidence is often constrained by methodological heterogeneity and varying risks of bias across studies.

### 6.4. Pharmacological Evidence

Metoclopramide and prochlorperazine are the most extensively studied dopamine receptor antagonists in the acute treatment of migraine, particularly for patients presenting with severe nausea, vomiting or intolerance to oral therapy. Beyond their antiemetic properties, these agents appear to exert a beneficial effect on headache intensity itself.

A randomized study compared intramuscular prochlorperazine, metoclopramide and placebo in 86 adult patients administered during a migraine attack [166]. At 60 min, pain intensity reduction was greater with prochlorperazine (67%) than with metoclopramide (34%) and placebo (16%); nausea and vomiting symptoms were also significantly improved in the prochlorperazine group. Consistent with these findings, another study observed a trend toward superior nausea relief with prochlorperazine, although the difference compared to metoclopramide and placebo did not reach statistical significance [167]. Beneficial effects on both pain and associated autonomic symptoms have also been reported for metoclopramide [168] and droperidol [169].

The therapeutic response to these agents should not be viewed as a definitive marker of a dopaminergic phenotype. Several other classes of acute treatments also alleviate migraine-associated symptoms. For instance, a comparative study between zolmitriptan and a combination of acetylsalicylic acid and metoclopramide found no significant differences in the reduction in nausea, vomiting, photophobia and phonophobia between the two groups [170]. Furthermore, a recent exploratory analysis from the PRODROME trial demonstrated that ubrogepant (a CGRP receptor antagonist) administered during the prodrome significantly improved symptoms such as photophobia, fatigue, neck stiffness and cognitive impairment compared to placebo [171].

Within the framework of the “dopaminergic phenotype”, these pharmacological observations are supportive but not pathognomonic. Symptomatic relief following the administration of metoclopramide or prochlorperazine does not unequivocally establish a dopaminergic etiology for an individual attack, as these agents exert pleiotropic effects and have predominantly been evaluated in unselected populations. Nevertheless, these findings underscore that for patients with a prominent gastrointestinal–autonomic component, dopamine receptor blockade remains a valid option of acute management.

### 6.5. Evidence Gaps and Future Directions

Despite the substantial reported prevalence of dopaminergic symptoms in migraine patients, validated clinical criteria and biological markers for a distinct dopaminergic phenotype are lacking. To date, no validated thresholds or scoring systems have been developed for classifying this phenotype or identifying a high dopamine-associated symptom burden. Existing studies typically classify the presence of even a single dopaminergic symptom as a dopaminergic presentation [54,63,156]. Such a broad definition is likely to have limited specificity, inflate prevalence estimates, and combine clinically heterogeneous presentations.

Another major challenge in defining a distinct dopamine-associated migraine phenotype is that the proposed symptoms frequently co-occur with established sensory, autonomic, and gastrointestinal features of migraine [59]. Moreover, the existing cross-sectional studies demonstrate substantial between-patient variation in the number and timing of dopamine-associated symptoms [5,59]. There is some evidence that individual dopamine-associated symptoms may recur across attacks [161,162,163,172]. However, no studies have assessed the stability and consistency of the dopamine-associated symptom profile between attacks. Dopamine receptor antagonists have demonstrated efficacy in the acute treatment of nausea, vomiting, and headache [150,152,157]. Nevertheless, no clinical trial has used a prespecified composite measure to determine whether dopaminergic treatments modify the broader dopaminergic profile or selectively benefit patients presenting with cluster of dopamine symptoms. Due to these limitations, the dopaminergic phenotype remains largely a symptom-based descriptive construct, without clear evidence of the advantage of using dopaminergic agents as a phenotype-directed pharmacological strategies.

Further research should initially establish a consensus symptom set and standardized assessment method. Prospective repeated-attack studies should then quantify within-patient stability, distinguish attack-related symptoms from interictal background symptoms, and evaluate whether reproducible symptom clusters can be identified. Overall, consistent definitions and outcome measures are needed to establish the clinical relevance and biological validity of the proposed dopaminergic phenotype.

## 7. Neuroimaging of the Dopaminergic System in Migraine

Neuroimaging represents the primary non-invasive approach for assessing the dopaminergic system in human migraine patients [173,174]. Research in this field typically follows two complementary axes: the direct examination of the dopaminergic system using radioligands targeting receptors or transporters, and the indirect assessment of structural or functional changes within CNS networks associated with dopaminergic signaling [88,173,174,175].

Direct molecular imaging, utilizing Positron Emission Tomography (PET) and Single-Photon Emission Computed Tomography (SPECT) remains the “gold standard” for the in vivo quantification of dopaminergic neurotransmission. Magnetic Resonance Imaging (MRI) provides proxy measures of dopamine circuits rather than direct physiological data [176,177,178]. In the migraine literature, the primary radioligands reported to date are [11C]raclopride and [123I]iodobenzamide (IBZM), both of which bind to D2 receptors (with [11C]raclopride also exhibiting affinity for D3 receptors) [88,179,180,181,182]. [18F]DOPA PET and dopamine transporter (DAT) imaging are well-established in the study of Parkinson’s disease and psychosis [183,184], but they have seen limited application in migraine research, with DAT-PET appearing in a single study on medication-overuse headache (MOH) [185].

Radiotracer-based approaches face operational challenges, including exposure to ionizing radiation and high costs. Consequently, most molecular imaging studies in migraine have been characterized by relatively small sample sizes [88,181,182,185]. Moreover, direct imaging studies employ complex protocols without a single standardized approach. Indirect methods, particularly MRI, offer substantial advantages, such as the lack of ionizing radiation and broader accessibility. However, the diversity of MRI modalities and the focus on varying regions of interest (ROIs) limit the comparability of findings across studies [175].

### 7.1. Direct Dopamine System Neuroimaging

Direct evidence from molecular neuroimaging remains sparse. In our review we identified six relevant publications [88,179,180,181,182,185]. Two of these are historical studies primarily focused on migraine pharmacotherapy rather than underlying pathophysiology (Table 2).

In one of the earliest investigations, Verhoeff et al. utilized [123I]-IBZM SPECT to assess a cohort overusing ergotamine (predating current MOH definitions). The authors found no significant differences in striatal [123I]IBZM uptake between “on-ergotamine” and “off-ergotamine” states, or relative to healthy controls [181]. This study suggested that ergotamine does not extensively occupy striatal D2 receptors and likely does not cross the blood–brain barrier in significant amounts. Similarly, Wöber et al. examined whether the prophylactic efficacy of flunarizine was associated with D2 receptor blockade. Their results indicated that flunarizine does not act via the striatal dopaminergic system, arguing against a substantial role for dopamine in migraine. However, as this study focused on drug binding potential, its findings contribute more to the pharmacology of flunarizine than to the direct understanding of migraine pathophysiology [182].

The most compelling neuroimaging evidence to date was provided by DaSilva et al. [88]. In this episodic migraine (EM) case–control study, patients underwent PET scans both interictally and during a spontaneous attack, the latter involving thermal pain challenge to induce allodynia. The authors observed reduced displacement of [11C]raclopride during the ictus, consistent with lower endogenous dopamine release. Furthermore, allodynic stimulation triggered a sudden reduction in [11C]raclopride uptake in the insula, interpreted as phasic DA release in response to an aversive stimulus. Migraine patients exhibited diminished dopaminergic activity during headaches but experienced large, phasic fluctuations in DA release, particularly associated with allodynia. Despite its rigorous methodology, the study’s small sample size (n = 8) and complex protocol limit its generalizability.

Subsequent research has sought to integrate these findings with functional networks. Kim et al. [179] combined direct and indirect imaging to assess reward system connectivity, finding altered signaling between the nucleus accumbens and the amygdala, potentially modulated by D2/D3 receptors. Marino et al. [180] applied machine learning to an aggregated PET dataset. Although partially relying on previously published data, they concluded that the dopaminergic signal in the anterior putamen is a key feature distinguishing migraine patient from healthy controls. Finally, Liu et al. [185] utilized DAT-PET in patients with medication-overuse headache (MOH), finding altered dopamine transporter availability. The lack of a chronic migraine group without MOH limits these conclusions to the biology of medication overuse.

The collective evidence from PET and SPECT studies indicates that direct dopaminergic imaging in migraine does not support a simple “deficiency” model. In episodic migraine, state-dependent fluctuations in dopamine release and disruptions in reward circuitry appear to predominate. In contrast, in migraine-related MOH, an additional signal of presynaptic DAT dysregulation within the medial orbitofrontal cortex suggests a reward/addiction circuitry model, potentially reinforcing medication-seeking behavior. These findings suggest that the dopaminergic system is involved both in the primary biology of migraine attack and in the processes driving chronification. However, due to the small, highly selected samples and the heterogeneity and complexity of the study protocols, which limit the power of statistical analyses and generalizability of the findings, these conclusions remain preliminary and require replication in larger cohorts using standardized and comparable methods.

### 7.2. Indirect Neuroimaging Studies Assessing Dopamine System Circuits and Structures

Over the past three decades, the increasing availability of functional magnetic resonance imaging (fMRI) has led to a surge in studies indirectly assessing the dopaminergic system and its functional connections. The earliest research focused on midbrain structures during the aura and ictal onset. Welch et al. [186] described a case of MwA where T2-weighted MRI during a spontaneous aura revealed increased signal intensity in the occipital cortex, as well as bilaterally in the red nucleus (RN) and substantia nigra (SN). This single-case study was historically significant because it was the first to suggest that midbrain nuclei, and not only cortical structures, are involved in the aura phase. Cao et al. subsequently expanded on these findings using BOLD-fMRI and repetitive checkerboard stimulation in a larger cohort [70].

More recent investigations have focused on the mesocorticolimbic reward system and complex network models involving interhemispheric, salience, default-mode, visual and limbic connectivity. OuYang et al. investigated interhemispheric connectivity in relation to symptom lateralization and migraine phenotypes. By correlating fMRI findings with neurotransmitter atlases, they found that clinical characteristics and headache laterality were associated with the spatial distribution of D1 receptors, D2 receptors, the norepinephrine transporter (NET) and GABA-A receptors [187]. The authors suggested the existence of distinct network-molecular subtypes within MwoA populations. However, dopaminergic activity was inferred from atlas-based measures rather than directly quantified in the study participants.

#### 7.2.1. The Midbrain and Brainstem

The hypothesis that midbrain structures, particularly the red nucleus (RN) and substantia nigra (SN), act as early nodes in migraine attacks evolved from historical imaging showing increased signal in these regions even before cortical changes or symptom onset [70,186]. Subsequent research shifted the focus to the premonitory phase; Maniyar et al. [188] observed activation of the posterolateral hypothalamus, midbrain tegmentum and periaqueductal gray (PAG) before the headache phase commenced.

Huang et al. [189] demonstrated altered functional connectivity between the RN/SN and somatosensory, frontal, and parietal regions in MwoA patients, with changes correlating with disease duration and HIT-6 scores. Additionally, transcranial sonography has revealed a higher frequency of hyperechogenicity in the SN and basal ganglia of migraineurs, particularly those with aura [190]. RN, SN and midbrain tegmentum are involved at the earliest stages of the ictal process and do not merely reflect a secondary response to pain.

#### 7.2.2. Basal Ganglia and Striatum

Another line of research investigates the role of the basal ganglia in migraine chronification, often using iron accumulation as a proxy for metabolic burden. Kruit et al. [191]. found lower T2 values (suggesting iron deposition) in the putamen, globus pallidus and RN, correlating with a longer history of migraine. However, follow-up data from the CAMERA cohort were more nuanced, suggesting that age-related microstructural changes might mask these effects over time [192].

Recent studies have highlighted structural differences across the frequency spectrum. Patients with high-frequency attacks exhibit attenuated pain-related responses in the caudate and putamen, alongside increased caudate volumes [193]. The nucleus accumbens (NAc) has also emerged as a key structure, showing increased iron deposition and hypoperfusion in chronic migraine [194,195]. Interestingly, Birkl et al. used a longitudinal single-subject design to show that iron-sensitive signals (R*2) exhibit short-term, reversible dynamics across the migraine cycle, suggesting that these changes fluctuate with individual attacks [196].

#### 7.2.3. The Reward System

Broad research has investigated the role of dopaminergic structures in addiction-like behaviors associated with medication-overuse headache (MOH). MOH can develop across various primary headache disorders, but its strong association with chronic migraine (CM) provides a model for studying reward and addiction circuitry in the context of migraine chronification [52,197].

Ferraro et al. identified a dual pattern in MOH patients during medication withdrawal: reduced activity in the SN and ventral tegmental area (VTA), paired with increased activity in the ventromedial prefrontal cortex (vmPFC) [198]. Notably, the dysregulation within the SN and VTA persisted after withdrawal, suggesting a deep-seated disruption of the reward system. Androulakis et al. demonstrated that both CM and MOH share a reduction in salience network connectivity, but MOH is characterized by more pronounced prefrontal-subcortical disruptions [199].

Even in episodic migraine (EM), dysfunction of reward and loss circuitry is present. Kocsel et al. [200] observed decreased activation of the right inferior frontal gyrus during reward consumption. Reward system impairment may already be present in the episodic phase, potentially acting as a substrate for future chronification.

#### 7.2.4. Summary of Indirect Neuroimaging Research

Studies implicate midbrain regions, the basal ganglia, striatal structures, the nucleus accumbens (NAc)-centered reward circuitry and prefrontal control systems in migraine pathophysiology. These findings align with clinical observations of dopaminergic symptoms, allodynia and medication-overuse headache (MOH), but they remain insufficient to confirm primary alterations in dopaminergic neurotransmission.

A critical consideration is that the examined regions exhibit different dopaminergic profiles, varying in receptor density and the functional role of dopamine. It is highly probable that dopamine interacts with multiple neurotransmitters and pain-modulatory networks to influence ictal states, aversive processing and reward dysregulation.

Future research should employ multimodal designs, combining molecular imaging (PET or SPECT) with functional MRI (fMRI). Furthermore, longitudinal phenotyping and precise attack-phase characterization are essential to distinguish whether observed circuit abnormalities reflect primary dopaminergic dysfunction, secondary adaptation to recurrent attacks or the neurobiological consequences of chronic medication overuse.

## 8. Migraine Comorbidities: A Dopaminergic Vulnerability

The hypothesis of a distinct dopaminergic phenotype in migraine is reinforced by its clinical association with other neurological and psychiatric disorders characterized by dopaminergic dysregulation. These comorbidities likely reflect shared genetic susceptibility.

An association is observed with restless legs syndrome (RLS), which shares a common substrate with migraine in the hypothalamic A11 nucleus [7,201]. Epidemiological data indicate that RLS is up to three times more prevalent in migraineurs than in the general population, particularly in those with frequent attacks. From a mechanistic perspective, both conditions converge on the hypothalamic A11 nucleus. As the primary source of dopaminergic projections to the spinal cord, the A11 nucleus acts as a gatekeeper for sensory processing [202]. In migraine, A11 dysfunction lowers the trigeminovascular threshold, while in RLS, it results in a failure to inhibit spinal somatosensory afferents, leading to the characteristic nocturnal motor symptoms. This “diencephalospinal” link suggests that a primary hypothalamic dopaminergic deficit may be a unifying trait for a subset of patients [203,204].

Furthermore, migraine shows significant comorbidity with ADHD and Tourette Syndrome, both of which involve dysregulation of the cortico-striato-thalamo-cortical loops [205]. In ADHD, altered dopamine transporter (DAT) density and impaired tonic dopamine release within the nucleus accumbens (NAc) mirror the reward system abnormalities described in chronic migraine and medication-overuse headache (MOH). This suggests that the “migraine brain” may share a reward-deficiency endophenotype, making these individuals more prone to sensory seeking during the prodrome (e.g., food cravings) and potentially increasing the risk of drug reinforcement in MOH [206,207].

Finally, the relationship between migraine and Parkinson’s disease (PD) provides a provocative insight into the dynamics of the dopaminergic system. Large-scale cohort studies, such as the AGES-Reykjavik study, have shown that individuals who experience migraine with aura in midlife have a significantly higher risk of developing parkinsonian symptoms later in life [208]. This does not necessarily imply a common neurodegenerative pathway but may point to a “long-term dopaminergic strain” [209]. The lifelong recurrence of dopaminergic fluctuations during migraine attacks characterized by ictal “crashes” and interictal instability might predispose the substantia nigra and related circuits to functional decline [210].

In summary, these comorbidities suggest that dopaminergic dysregulation in migraine is not an isolated ictal phenomenon but a manifestation of a systemic neurobiological trait. This “dopaminergic vulnerability” spans from the hypothalamus to the basal ganglia, shaping a complex clinical landscape that extends far beyond the headache itself.

## 9. Conclusions

The role of the dopaminergic system in migraine pathophysiology is a complex, multifaceted phenomenon that transcends simplified models of neurotransmitter deficiency or excess. This review shows that dopamine functions primarily as a modulator of the trigeminovascular threshold, influencing the expression of premonitory symptoms and the trajectory of pain chronification. A central hub of this regulation appears to be the hypothalamic A11 nucleus, which integrates nociceptive signaling with autonomic responses through the concentration-dependent activation of D1 and D2 receptors.

Neuroimaging and genetic evidence support the existence of a distinct “dopaminergic phenotype”, characterized by prominent nausea, yawning and reward system dysfunction, and this framework is further validated by the clinical co-occurrence of migraine with other dopamine-related disorders, such as Restless Legs Syndrome and ADHD.

Future research utilizing multimodal imaging and sex-stratified analyses across various hormonal states is essential to fully elucidate the nuanced role of dopamine. Ultimately, such insights will facilitate a paradigm shift from empirical symptomatic management toward personalized, mechanism-based therapeutic strategies.

## Figures and Tables

**Table 1:** Dopaminergic symptoms across migraine phases and their proposed pathophysiological mechanisms.

Structures Involved	Pathophysiological Mechanism	Clinical Significance	Dopaminergic Symptoms	Migraine Phase
D2/D3 receptors and hypothalamus (A11 nucleus) [36]	Receptor hypersensitivity triggered by low interictal DA levels and early hypothalamic activation [59]	Yawning (63.6%) and somnolence (57.5%) are highly prevalent, with a high predictive value for the upcoming headache [59]	Yawning, somnolence, nausea, mood fluctuations and polyuria [68]	**Prodrome (premonito** **ry)**
D1 receptors (pro-nociceptive), A11-TCC axis and area postrema [37,92]	Ictal DA surge activating low-affinity pro-nociceptive pathways and stimulation of the area postrema [59]	Nausea is reported by 73% of patients and often ranked as the most bothersome symptom [157]	Nausea, vomiting, hypotension and cutaneous allodynia [68]	**Headache**
D1/D2 receptors, prefrontal cortex, and striatum [59,126]	Post-ictal dopamine depletion and downregulation of DA receptors [59]	Reported by 80% of patients, fatigue (55–88%) and cognitive difficulties predominate [27]	Fatigue, somnolence, cognitive impairment (“brain fog”), mood fluctuations and hunger [68]	**Postdrome**

**Table 2:** Characteristics of dopaminergic imaging studies in migraine.

Main Findings	Imaging Method (and Target)	Imaging Phase	Phenotype (Population)	Study
No significant difference in striatal D2 binding between ergotamine use, withdrawal and HC	[123I]IBZM SPECT (striatal D2 BP)	Not specified	MwoA + Ergotamine overuse (n = 2) and HC (n = 15)	**Verhoeff et al., 1993** [181]
Flunarizine reduced D2 BP but the degree of blockade did not correlate with prophylactic efficacy	[123I]IBZM SPECT (striatal D2 BP)	Interictal	Migraine + Flunarizine (n = 11) and HC (n = 21)	**Wöber et al., 1994** [182]
Striatal BPnd increased during ictus/allodynia, indicating reduced endogenous DA release. Phasic fluctuations in the insula were observed	[11C]raclopride PET (D2/D3 receptor availability and endogenous dopamine release)	Ictal vs. interictal	EM (n = 8) and HC (n = 8)	**DaSilva et al., 2017** [88]
Weaker right Nac-amygdala connectivity was inversely associated with D2/D3 receptor availability and related to lower interictal positive effect and greater ictal pain severity	rs-fMRI + [11C]raclopride PET (D2/D3 receptor availability and NAc-centered circuitry)	Interictal	EM (n = 11) and HC (n = 10)	**Kim et al., 2021** [179]
ML classified migraine vs. HC with >90% accuracy and the anterior putamen was identified as the most informative D2/D3 region	PET-based compressive big data analytics using [11C]raclopride and [11C]carfentanil datasets (D2/D3 predictive pattern)	Ictal vs. interictal	EM (n = 31), CM (n = 7) and HC (n = 23)	**Marino et al., 2023** [180]
Reduced DAT availability in the medial OFC in MOH, suggesting dysregulation in reward/addiction circuitry	[11C]CFT PET/MR (DAT availability in the medial OFC)	Not specified	MOH with migraine history (n = 17) and HC (n = 16)	**Liu et al., 2024** [185]

**Abbreviations:** BP—binding potential; BPnd—non-displaceable binding potential; CFT—2β-carbomethoxy-3β-(4-fluorophenyl)tropane; CM—chronic migraine; D2/D3—dopamine D2/D3 receptors; DA—dopamine; DAT—dopamine transporter; EM—episodic migraine; fMRI—functional magnetic resonance imaging; HC—healthy controls; IBZM—iodobenzamide; MR—magnetic resonance; MOH—medication-overuse headache; MwA—migraine with aura; MwoA—migraine without aura; Nac—nucleus accumbens; OFC—orbitofrontal cortex; PET—positron emission tomography; rs-fMRI—resting-state functional magnetic resonance imaging; SPECT—single-photon emission computed tomography.

## Data Availability

No new data were created or analyzed in this study. Data sharing is not applicable to this article.

## Abbreviations

The following abbreviations are used in this manuscript:

5-HT	5-hydroxytryptamine (serotonin)
AADC	Aromatic L-amino acid decarboxylase
ADHD	Attention-deficit/hyperactivity disorder
AMPP	American Migraine Prevalence and Prevention
BP	Binding potential
BPnd	Non-displaceable binding potential
CGRP	Calcitonin gene-related peptide
CM	Chronic migraine
CNS	Central nervous system
COMT	Catechol-O-methyltransferase
CSD	Cortical spreading depression
DA	Dopamine
DALYs	Disability-adjusted life years
DAT	Dopamine transporter
DBH	Dopamine beta-hydroxylase
DOPAC	3,4-Dihydroxyphenylacetic acid
DRD	Dopamine receptor
EM	Episodic migraine
FHM	Familial Hemiplegic Migraine
fMRI	Functional magnetic resonance imaging
GABA	Gamma-aminobutyric acid
GWAS	Genome-wide association studies
HC	Healthy controls
IBZM	Iodobenzamide
ICHD-3	International Classification of Headache Disorders, 3rd edition
L-DOPA	L-3,4-dihydroxyphenylalanine
MAOA	Monoamine oxidase A
ML	Machine learning
MOH	Medication-overuse headache
MRI	Magnetic resonance imaging
MwA	Migraine with aura
MwoA	Migraine without aura
NAc	Nucleus accumbens
NE	Norepinephrine
NGS	Next-Generation Sequencing
NSAID	Non-steroidal anti-inflammatory drug
OFC	Orbitofrontal cortex
Oct	Octopamine
PAG	Periaqueductal gray
PAH	Phenylalanine hydroxylase
PEA	Phenylethylamine
PET	Positron emission tomography
PNMT	Phenylethanolamine N-methyltransferase
RLS	Restless legs syndrome
RN	Red nucleus
ROI	Region of interest
ROS	Reactive oxygen species
SLC6A3	Solute carrier family 6 member 3
SN	Substantia nigra
SPECT	Single-photon emission computed tomography
Syn	Synephrine
TA	Trace amine
TAAR	Trace amine-associated receptor
TCC	Trigeminocervical complex
TH	Tyrosine hydroxylase
TNC	Trigeminal nucleus caudalis
TRPV1	Transient receptor potential vanilloid 1
TTH	Tension-type headache
Tyr	Tyramine
vmPFC	Ventromedial prefrontal cortex
VTA	Ventral tegmental area
